# Revealing the characteristics of a novel bioflocculant and its flocculation performance in *Microcystis aeruginosa* removal

**DOI:** 10.1038/srep17465

**Published:** 2015-12-02

**Authors:** Pengfei Sun, Cai Hui, Naling Bai, Shengmao Yang, Li Wan, Qichun Zhang, YuHua Zhao

**Affiliations:** 1 College of Life Sciences, Zhejiang University, 310058 Hangzhou, Zhejiang, PR China; 2 Institute of Environment Resource and Soil Fertilizer, Zhejiang Academy of Agriculture Science, 310021 Hangzhou, Zhejiang, China; 3 Department of Chemical and Biomolecular Engineering, Vanderbilt University, 37235-1604, Nashville, Tennessee, USA; 4 College of Environmental and Resource Sciences, Zhejiang University, 310058 Hangzhou, Zhejiang, PR China

## Abstract

In the present work, a novel bioflocculant, EPS-1, was prepared and used to flocculate the kaolin suspension and *Microcystis aeruginosa*. We focused on the characteristics and flocculation performance of EPS-1, especially with regard to its protein components. An important attribute of EPS-1 was its protein content, with 18 protein types identified that occupied a total content of 31.70% in the EPS-1. Moreover, the flocculating activity of these protein components was estimated to be no less than 33.93%. Additionally, polysaccharides that occupied 57.12% of the total EPS-1 content consisted of four monosaccharides: maltose, D-xylose, mannose, and D-fructose. In addition, carbonyl, amino, and hydroxyl groups were identified as the main functional groups. Three main elements, namely C1s, N1s, and O1s, were present in EPS-1 with relative atomic percentages of 62.63%, 24.91%, and 10.5%, respectively. Zeta potential analysis indicated that charge neutralization contributed to kaolin flocculation, but was not involved in *M. aeruginosa* flocculation. The flocculation conditions of EPS-1 were optimized, and the maximum flocculating efficiencies were 93.34% within 2 min for kaolin suspension and 87.98% within 10 min for *M. aeruginosa*. These results suggest that EPS-1 could be an alternative to chemical flocculants for treating wastewaters and cyanobacterium-polluted freshwater.

Sources of clean freshwater are crucial for sustaining human life. However, many freshwater sources have become contaminated by harmful cyanobacteria and are thus unsafe for human use. Cyanobacterial pollution poses a grave problem not only to regional economic development but also to the safety of aquatic animals and humans because many harmful cyanobacteria, such as *Microcystis aeruginosa* (notorious for causing harmful cyanobacteria blooms in freshwater), produce toxins, including neurotoxins and hepatotoxins^1^,^2^. Therefore, there is an urgent need to explore feasible approaches for the treatment of harmful cyanobacteria pollution which has resulted in the development of various methods for decreasing its negative effects. Among these, flocculation is a preferred method because of its high efficiency and simplicity^3^. Flocculants can be classified into three groups: inorganic flocculants (polyaluminum chloride and aluminum sulfate), organic synthetic flocculants (polyethylene imine and polyacrylamide derivatives), and naturally occurring flocculants (chitosan, tannins, and bioflocculants)^4^,^5^,^6^,^7^. Currently, various chemical flocculants, including polyaluminum chloride, aluminum sulfate, and polyacrylamide are being applied in industrial processes such as wastewater treatment, downstream processing of biopharmaceutical proteins, dehydration of activated sludge, production of tap water, dredging, and fermentation. However, the extensive use of chemical flocculants has been restricted because of their neurotoxic and carcinogenic properties^8^. With the restriction of the use of chemical flocculants, there is a threat to the use of flocculation technology, necessitating the identification of new alternatives.

As an alternative to chemical flocculants, bioflocculants have been widely studied in recent years as a promising option for wastewater treatment because of their improved efficiency, innocuity, and biodegradability compared with that of traditional flocculants^9^,^10^,^11^,^12^,^13^,^14^. Since 1984 when Fattom and Shilo found that *Phormidium* J-1 could produce a polymer to flocculate bentonite^15^, many microorganisms have been studied for their ability to produce bioflocculants. For example, *Bacillus* sp. AEMREG7 was shown to produce a bioflocculant with a maximum flocculating activity of 92.6% against kaolin clay suspension^16^, and *Enterobacter cloacae* sp. WD7 and *Pseudomonas alcaligenes* WD22 were shown to produce bioflocculants with flocculating activities of 91% and 55% , respectively^17^. Fungi such as *Aspergillus parasiticus* was shown to produce a bioflocculant with a flocculating activity of 92.4% against Reactive Blue 4, and an *Aspergillus niger* bioflocculant had a flocculating activity of 63% for turbidity removal^18^,^19^. Actinomyces such as *Nocardia* spp. were shown to produce a highly efficient bioflocculant^20^, and algae such as *Desmodesmus* sp. F51 were found to produce a bioflocculant (Poly-γ-glutamic acid) with a flocculating activity of 96%^21^. Consequently, the screening of new strains producing highly efficient bioflocculants with reduced production cost became an important research topic in this field^5^,^22^. In addition, the application of bioflocculants in the control of harmful algae blooms has great practical significance. However, to the best of our knowledge, there are no reports on the treatment of *M. aeruginosa* pollution using *Bacillus amyloliquefaciens* bioflocculants.

In this study, we identified a novel bioflocculant and investigated its characteristics including protein components and flocculation activity. Response surface methodology (RSM) was used to optimize the parameters of the bioflocculant for treating kaolin suspension and *M. aeruginosa* pollution in freshwater. The results of our study suggest that this bioflocculant offers a highly efficient solution for the treatment of *M. aeruginosa* pollution.

## Results and Discussion

### Isolation and identification of Bacillus amyloliquefaciens DT

**Figure 1:**
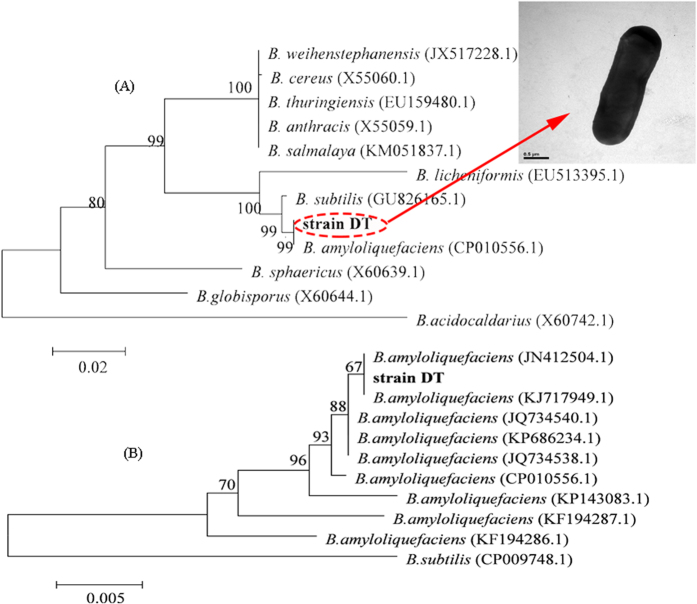
Identification of strain DT. (**A**) A transmission electron microscopic image of strain DT (the scale bar = 0.5 μm), and a neighbor-joining phylogenetic tree of strain DT and other related species based on 16S rDNA sequences. Bootstrap values (percentages of 1,000 replications) are shown at the branch points. The scale bar = 0.02 substitutions per nucleotide position (evolutionary distance). (**B**) A neighbor-joining phylogenetic tree of strain DT and other related species based on *gyrB* sequences. Bootstrap values (percentages of 1,000 replications) are shown at the branch points. The scale bar = 0.005 substitutions per nucleotide position (evolutionary distance).

A bioflocculant-producing bacterium was chosen for further study and named as strain DT. Strain DT was originally isolated from restaurant garbage and is currently preserved at the China General Microbiological Culture Collection Center (CGMCC). Its registration number is 9196; thus, it is known as CGMCC No. 9196. An alignment of 16S rDNA sequences and a phylogenetic tree (presented in Fig. 1A) indicated that strain DT had 100% similarity to *Bacillus amyloliquefaciens*. Furthermore, an alignment of *gyrB* sequence (1237 bp, shown in Fig. S1) and a phylogenetic tree (presented in Fig. 1B) indicated that strain DT had 100% sequence similarity to *B. amyloliquefaciens*, the strain was therefore named as *Bacillus amyloliquefaciens* DT. Additionally, strain DT was observed as a rod-shaped bacterium without flagella, and had a size of approximately 0.5 × 2.25 μm (Fig. 1A). In terms of its functions, *B. amyloliquefaciens* has generally been proven to be a useful biocontrol strain in agriculture^23^. Moreover, in a currently unpublished study, we found that strain DT was a highly efficient starch-degrading bacterium, which shows strong potential for the treatment of restaurant garbage. Therefore, with the addition of its observed flocculating property, the three functions of *B. amyloliquefaciens* DT make it a multifunctional bacterium that could be applied for the biocontrol of agricultural pests, biodegradation of restaurant garbage, as well as the control of harmful cyanobacterial blooms.

### Characteristics of EPS-1

#### Chemical analysis of EPS-1

**Figure 2:**
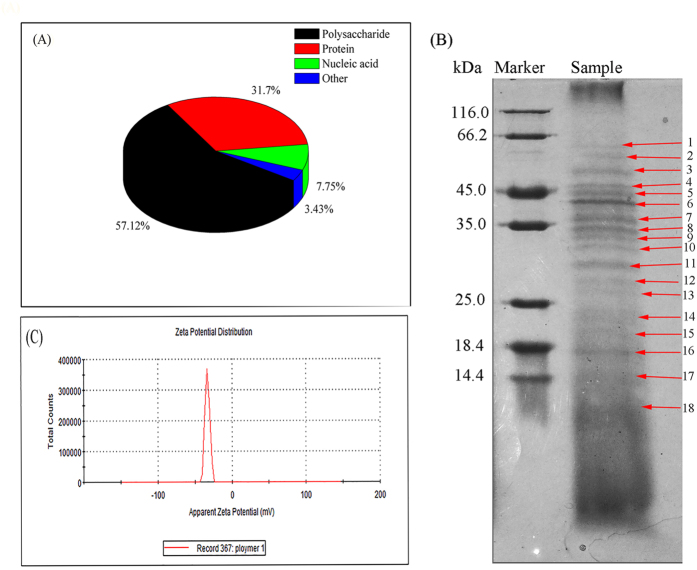
Characteristics of the bioflocculant EPS-1. (**A**) Chemical composition analysis. (**B**) Protein profiles of the EPS-1 solution: the numbers (1–18) denote the different protein types; (**C**) Diagram of zeta potential of EPS-1 solution.

The yields of bioflocculants were in the range of 0.126–3.58 g/L^18^,^24^,^25^,^26^. A total of 0.36 g of purified EPS-1 was obtained from 1 L of fermentation broth, indicating that the yield of the purified ESP-1 is acceptable. The chemical composition of purified EPS-1 was assessed, and the results showed that the polysaccharide and protein content were 57.12% and 31.70%, respectively; moreover, the nucleic acid content, which is an indicator of cell lysis, was 7.75% (Fig. 2a). In contrast, the protein content in this bioflocculant was remarkably higher than that of other reported biopolymers; for instance, the protein contents of the biopolymers ZZ-3 and EPS were only 6.1%^26^ and 16.41%^27^, respectively. In addition, unidentified substances (impurities) accounted for 3.43% of the total content of the purified EPS-1.

#### Monosaccharide, protein composition, and molecular weights analysis

High performance liquid chromatography (HPLC) was used to identify the monosaccharide composition of EPS-1, with retention times being compared to those of known standards. The results indicated that EPS-1 primarily consists of maltose (10.53 min), D-xylose (13.133 min), mannose (16.237 min), and D-fructose (16.882 min).

The molecular weights and diversities of proteins in EPS-1 were detected by SDS-PAGE analysis. Figure 2b shows that there are at least 18 types of different proteins in EPS-1, which were identified according to their different molecular weights, and the molecular weights of the majority of proteins were 18.4–66.2 kDa. These results suggest that the types of proteins in EPS-1 are considerably complex. Combined with the results of monosaccharide composition analysis, these findings indicate the complex composition of EPS-1.

#### Zeta potential analysis

The electric charge on the surface of the EPS-1 monomer solution was measured at pH 2.08 at 25 °C. Although the zeta potentials of most of the bioflocculants were negative^28^,^29^, the negative zeta potential value of EPS-1 (−33.67 ± 0.90 mV, Fig. 2c) was substantially different from the positive values of PSB-1, PSB-2, and PSB-3^22^ and the positive values of poly-γ-glutamic acid at pH 3.0^21^. In general, the zeta potentials of acidic proteins are usually negative. We therefore speculate that the negative zeta potential of EPS-1 is related to its abundance of proteins or acidic proteins.

#### Contribution of proteins to flocculating activity of EPS-1

**Figure 3:**
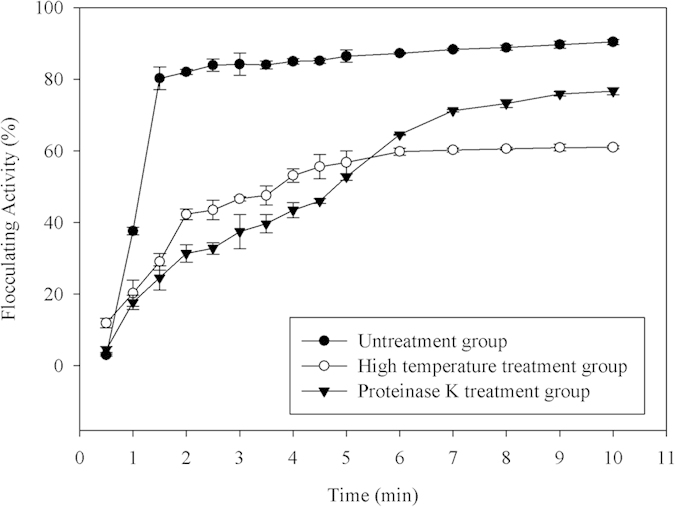
Comparisons of the flocculating activities in untreated EPS-1 solution and solutions treated with either high temperature or proteinase K.

Because the total protein content of the EPS-1 is up to 31.70%, we were interested in understanding the contribution of the proteins to the total flocculating activity (Fig. 3). To our knowledge, polysaccharides are tolerant to high temperatures and proteinase K, whereas most proteins are susceptible to these treatments. The flocculating activity of untreated EPS-1 against kaolin suspension was 92.31 ± 0.75%; when EPS-1 was subjected to high temperature (100 °C) and proteinase K treatments, the corresponding flocculating activities decreased to 60.99 ± 0.48% and 76.68 ± 0.99%, respectively (Fig. 3). Therefore, the contribution of proteins to the total flocculating activity of EPS-1 was estimated to be no less than 33.93% [(92.31 − 60.99)/92.31 × 100% = 33.93%], which is in agreement with the total protein content in EPS-1.

#### Fourier transform infrared (FTIR) analysis

**Figure 4:**
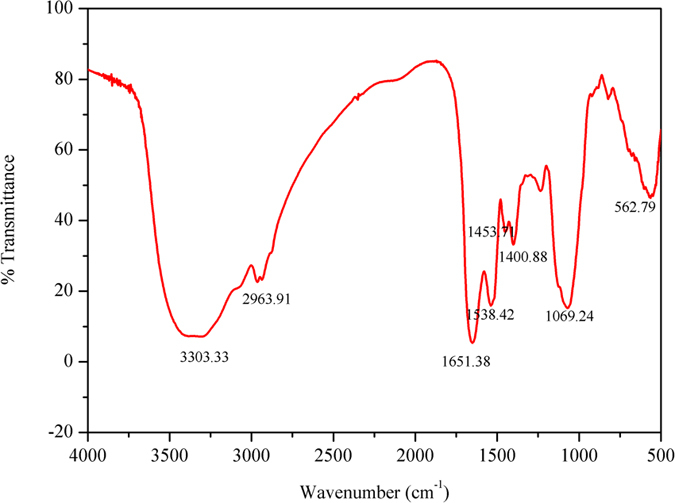
Fourier transform-infrared spectrograms of the bioflocculant EPS-1.

The functional groups of EPS-1 were analyzed using FTIR spectroscopy; the spectra at 4000–500 cm^−1^ are reproduced in Fig. 4. The results showed a strong absorption peak at 3303.33 cm^−1^ generated by the stretching vibration of –OH or –NH groups, and a weak C–H stretching band represented by the peak at 2963.91 cm^−1^. Bands at 1651.38 and 1538.42 cm^−1^ represent the functional group of carbonyl, and the absorption band at 1650 cm^−1^ is associated with the characteristic vibrations of the C = O stretching in the –CONH– group in proteins and amino-sugars^22^; the second strong peak suggested that the relative content of proteins and amino sugars was high, indicating that proteins or amino sugars could play an important role in the flocculation process. A weak symmetrical stretching peak at 1403 cm^−1^ shows the presence of carboxyl groups. The peaks around 1000–1100 cm^−1^ were reported as the characteristic peaks of all sugar derivatives^30^. Both weak and strong adsorption peaks at 1234.48 and 1069.24 cm^−1^, respectively, were related to C-O stretching and provide further evidence to support the presence of methoxyl groups^26^. In conclusion, the infrared spectra showed characteristic functional groups that mainly included carbonyl, amino, and hydroxyl groups and amides, which indicate that EPS-1 is primarily a mixture of hetero-polysaccharide and proteins.

#### Elemental analysis

**Figure 5:**
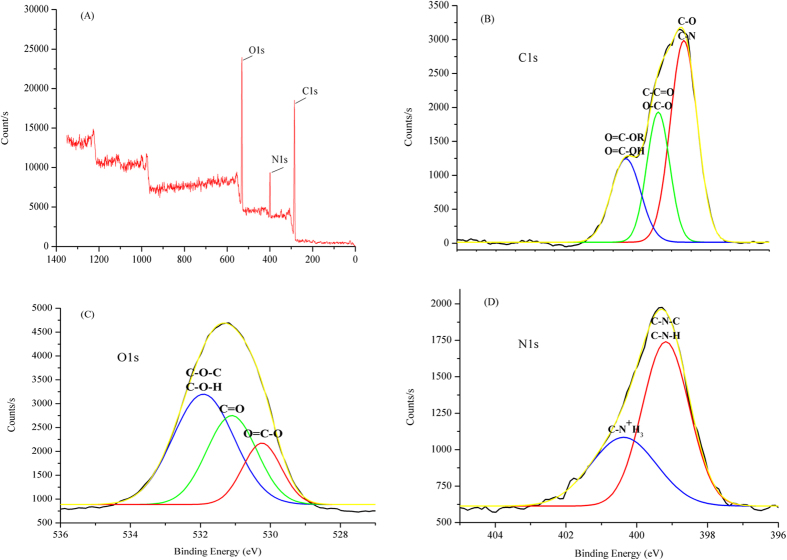
XPS analysis of the atomic composition of the bioflocculant EPS-1.

The total element types and their relative contents in the EPS-1 were determined by using a full-scan spectrum of X-ray photoelectron spectrometer (XPS) analysis. The results, shown in Fig. 5a, indicated that there are three main atomics in EPS-1, namely C1s, O1s, and N1s. The atomic relative percentages of the three elements were 62.63% for C1s, 24.91% for O1s, and 10.5% for N1s. Other minor elements were also detected; for instance, P2p (0.71%), F1s (0.54%), Cl2p (0.48%), and S2p (0.23%). In additional analyses, the chemical valences of these three main elements were re-determined by XPS. High-resolution scans of C1s, O1s, and N1s were deconvoluted into different valences to predict the potential functional groups (Fig. 5b–d). Results shown in Fig. 5b indicate that the C1s peak was resolved into three different peaks: the lowest peak at 287.38 eV is attributed to carboxyl or ester groups; the peak at 285.68 eV is likely associated with C = O or O-C-O groups from carboxylate, carbonyl, amide, or acetal; and the peak at 284.38 eV, which is associated with the group C-(C, H) from EPS-1, presents the largest percentage among the spectral bands. The peak of O1s was also deconvoluted and three peaks were generated (Fig. 5c): the largest peak at 531.88 eV is attributed to groups of C-O-(H, C) in acetal or ester; the peak at 531.08 eV is associated with O = C in carboxylate, carbonyl, ester, or amide; and the peak at 530.28 eV is probably produced by O = C-O from carboxylate. Two different forms of nitrogen composed the N1s peak (Fig. 5d): the peak at 400.38 eV is generated by protonated amines, being regarded as the fragment in amino-sugars, whereas the peak at 399.18 eV is non-protonated nitrogen from amines and amides. The biopolymers PSB-2 and PSB-3 produced from biological sludge and a composite bioflocculant produced by *Rhizobium radiobacter* F2 and *Bacillus sphaeicus* F6 mainly contain C and O^22^,^24^,^30^, whereas another bioflocculant produced by *Bacillus licheniformis* X14 is mainly composed of N and O^31^.Compared to these bioflocculants, EPS-1 was different in its atomic composition. These results suggest that there are many functional groups containing C, N, and O atoms that are preferred for flocculation, and these results were in agreement with those of the IR analysis and provide further evidence for the existence of carbonyl, amino, hydroxyl, and amide groups in the bioflocculant EPS-1.

### Optimization of flocculation parameters of EPS-1 for kaolin suspension and M. aeruginosa removal

Our preliminary experiments showed that three parameters, the dosage of EPS-1, pH, and CaCl_2_ content, are crucial for achieving a high flocculating capacity against kaolin suspension and *M. aeruginosa* cells. Thus, we investigated the interaction between these factors and optimized them by 3-level-3-factor BBD analysis in an attempt to obtain higher flocculation efficiencies against kaolin suspension and *M. aeruginosa* cells. The corresponding BBD and experimental data are shown in Table 1. The final models for kaolin suspension flocculating activity (Y_1_) and *M. aeruginosa* flocculating activity (Y_2_), derived using a Hetero–Fenton process, are given below (in terms of coded factors).

For kaolin suspension:


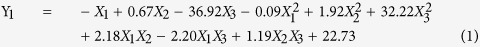


and for *M. aeruginosa*:


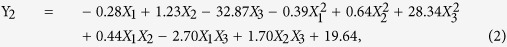


where Y_1_ and Y_2_ are the flocculating activities (%), and *X*_*1*_, *X*_*2*_, and *X*_*3*_are the CaCl_2_ content (g/L), pH value, and the dosages of EPS-1 (g/L), respectively.

The analysis of variance (ANOVA) results for the quadratic polynomial model shown in Table 2 strongly support the two models, with high model F-values (230.63 for Eq. 1 and 569.72 for Eq. 2) and low p values (p < 0.0001). The high R^2^ values (0.9976 for kaolin suspension and 0.9990 for *M. aeruginosa*) indicate a good agreement between the experimental and predicted values in this work. For flocculating kaolin suspension, the “pred-R^2^” of 0.9919 in the design is comparable with the “adj-R^2^” of 0.9933, and the value of adj-R^2^ (0.9933) suggests that 99.33% of the total variation in the flocculating process is attributable to the independent variables and that the model cannot explain only approximately 0.67% of the total variation. For flocculating *M. aeruginosa*, the “pred-R^2^” of 0.9868 in the design is also comparable with the “adj-R^2^” of 0.9973, and the value of adj-R^2^ (0.9973) suggests that 99.73% of the total variation in the flocculating process is attributable to the independent variables and that the model cannot explain only approximately 0.27% of the total variation. These results indicate that the two models are each suitable for describing the respective relationships between flocculation efficiency and the significant factors.

RSM has been widely used to optimize flocculation parameters. For example, Zhao *et al.* employed RSM to optimize flocculation parameters against a kaolin suspension and *Acanthamoeba cysts*^3^. In addition, RSM was also used to optimize flocculation parameters against a kaolin suspension and *M. aeruginosa* cells in our previous study^27^. Based on the results of BBD, the optimal parameters calculated from the regression equations were 53.87 mg/L EPS-1 dosage, 5.04 g/L CaCl_2_, and pH 2.08 for kaolin suspension removal, and 243.70 mg/L EPS-1 dosage, 5 g/L CaCl_2_, and pH 4 for *M. aeruginosa* removal. Among these three factors, EPS-1 is the core component for flocculating kaolin particles or *M. aeruginosa* cells. Metal ions are important for enhancing the flocculating activity of cation-dependent bioflocculants. Therefore, the Ca^2+^ ions provided by CaCl_2_ improved the flocculating activity of EPS-1 against kaolin particles and *M. aeruginosa* cells by neutralizing and stabilizing the negative charge of those functional groups, thus indicating the occurrence of a bridging mechanism during the flocculating process^19^,^32^; pH is also an important parameter because it can affect the surface charge and electrification state of bioflocculants and colloidal particles and thus have a great influence on the flocculating activity.

### Optimal flocculating properties of EPS-1 against kaolin suspension and M. aeruginosa

**Figure 6:**
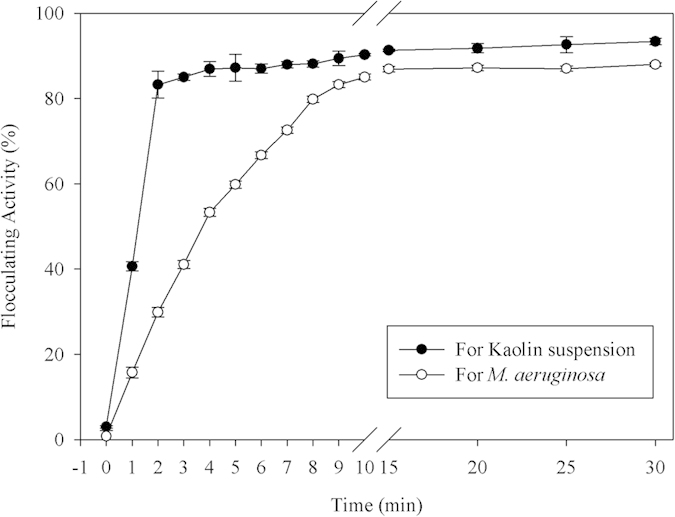
Flocculating activity of the bioflocculant EPS-1 against kaolin suspension and *M. aeruginosa*, given the optimal parameters. The data represent averages of three independent experiments, with ± SD indicated by error bars.

The flocculating efficiencies for kaolin suspension and *M. aeruginosa* were detected under optimal flocculating conditions. Figure 6 shows the high flocculating efficiencies against both kaolin suspension and *M. aeruginosa*. Both the flocculating activities rapidly achieved equilibrium for kaolin suspension (within 2 min) and *M. aeruginosa* (within 10 min), and the maximum flocculation efficiencies under optimal flocculation parameters were 93.34% within 2 min for kaolin suspension and 87.98% within 10 min for *M. aeruginosa*. In addition, the actual flocculating effect against kaolin suspension and *M. aeruginosa* culture was visualized, and these results are shown in Fig. S2. The flocculating activity of the bioflocculant EPS-1 compares favorably with the activities of other flocculants; for instance, the maximum flocculating efficiency of a modified sand flocculant for *M. aeruginosa* was 90% within 30 min^33^, whereas an activated fly ash-modified chitosan flocculant required 60 min to remove 90% of *M. aeruginosa*^34^. Therefore, our results suggest that the novel bioflocculant EPS-1 is highly efficient in removing considerable quantities of kaolin suspension and high concentrations of *M. aeruginosa* in a relatively short time.

To date, many studies on the microbial production of flocculating substances have been reported from different viewpoints^9^,^10^,^11^,^12^,^13^,^14^. Our study was mainly focused on the characteristics of EPS-1 and its flocculating activity against kaolin suspension and harmful *M. aeruginosa*. Our results showed that EPS-1 is highly efficient in the removal of kaolin clay and *M. aeruginosa* within a short time; therefore, this bioflocculant offers a new option for the treatment of wastewater containing strong acids and harmful cyanobacterial blooms, which is the subject of our ongoing investigation. In addition, we aim to overcome the challenge of enhancing the flocculating activity of EPS-1 against *M. aeruginosa* in real *M. aeruginosa*-polluted freshwater.

### Flocculating mechanisms of EPS-1 against kaolin suspension and M. aeruginosa

In further analyses, the zeta potentials of blank kaolin clay solution, blank *M. aeruginosa* culture, kaolin flocculating system solution, and *M. aeruginosa* flocculating system solution were determined to predict whether charge neutralization plays an important role in either of the two flocculating systems. As shown in Table 3, after flocculation with EPS-1, the zeta potential of the kaolin solution decreased markedly (from 18.5 ± 0.98 to 6.13 ± 0.29 mV), whereas the zeta potential of the *M. aeruginosa* culture showed little change (from −22 ± 5.16 to −21.6 ± 2.91 mV). This result indicates that charge neutralization plays an important role in the kaolin flocculation process, but that it was not involved in the *M. aeruginosa* flocculation process.

## Materials and Methods

### Isolation and identification of Bacillus amyloliquefaciens DT

Different strains of bacteria, which were previously enrichment-cultured from restaurant garbage samples collected from a canteen at Zhejiang University (Hangzhou, China), were screened for bioflocculant-producing bacteria in a screening medium^27^. The bacteria were cultured in the screening medium at 30 °C in a rotary shaker at 340 × *g* for 48 h. Kaolin suspensions (10 g/L) were then used to evaluate the flocculating activity of bacterial culture broths (the detailed method was described in the section ‘*Measurement of flocculating activity’*), and the strain with the highest flocculating efficiency, namely strain DT, was selected for further investigation. A Bacterial Genomic DNA Miniprep Kit (Axygen, USA) was employed to the extract the genomic DNA of this strain, which was then used as the template for 16 S rDNA and *gyrB* genes amplification. Primers used in the 16S rDNA amplification were 27F and 1492R^27^, and primers used in the *gyrB* gene amplification were *gyrB*-F (5′-GAA GTC ATC ATG ACC GTT CTG CAY GCN GGN GGN AAR TTY GA-3′) and gyrB-R (5′-AGC AGG ATA CGG ATG TGC GAG CCR TCN ACR TCN GCR TCN GTCAT-3′). The *gyrB* gene amplification process was conducted in a 50-μL volume on a MJ PTC-200 cycler (Bio-Rad) using Taq Master Mix (Takara). The following cycling parameters were used: 94 °C for 5 min; 30 cycles of 94 °C for 30 s, 58 °C for 30 s, and 72 °C for 2 min; and final extension at 72 °C for 5 min. The amplified genes were sequenced by Sangon Biotech Co., Ltd. (Shanghai, China), and then the sequences of strain DT were aligned with corresponding sequences from related organisms, which were retrieved from the GenBank database using the BLAST algorithm. Clustal X software was used to perform sequences alignment^35^, and neighbor-joining phylogenetic trees were then constructed using BioEdit and Mega 4.0^36^. Additionally, the morphological characteristics of strain DT were observed by using a JEM-2010 (High Revolution) transmission electron microscope (JEOL, Japan) with an accelerating voltage of 200 kV^37^.

### Extraction and purification of the bioflocculant

A modified extraction method was used to extract the bioflocculant^27^. Strain DT was initially cultured in the screening medium at 30 °C with oscillation at 340 × *g* for 48 h, and then the culture broth was centrifuged at 7200 × *g* for 30 min, and the supernatant was subsequently collected and treated as follows. Pre-cooled (−20 °C) absolute ethyl alcohol was added to the supernatant at twice the volume of the supernatant. The mixture was stabilized at 4 °C for 24 h, after which it was centrifuged and the precipitate was dried to produce the crude bioflocculant, which was named as EPS-1. The crude EPS-1 was dissolved in water, and an equal volume of Sevage solution (chloroform:*n*-butanol = 5:1) was then added and oscillated at a speed of 340 × *g* for 30 min. Afterwards, the mixture was centrifuged at 7200 × *g* for 30 min, and the supernatant was evaporated by heating (60 °C), which yielded purified EPS-1.

### Measurement of flocculating activity

To measure the flocculating activity of strain DT (FA_1_), 30 mL kaolin suspension (10 g/L), 1 mL fermentation broth, and 1 mL CaCl_2_ solution (10 g/L) were mixed in a 100 mL beaker. The reaction vessel was rapidly mixed (340 × *g*) for 2 min before the optical density (OD) at 550 nm of the upper phase was measured using a spectrophotometer (7230G, Shanghai, China) for 30 min. FA_1_ was calculated according to Eq. 3. Kaolin suspension mixed with 1 mL CaCl_2_ solution under the same conditions described above was used as the control.

To measure the flocculating activity of purified EPS-1 (FA_2_), 0.05 g of EPS-1 was dissolved in 1 L distilled water to prepare the bioflocculant solution. The aforementioned method was used to determine the flocculating activity of EPS-1, except that 1 mL of fermentation broth was replaced with 1 mL of bioflocculant solution. FA_2_ was also calculated according to Eq. 3.


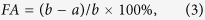


where *a* and *b* are the OD_550_ of the sample and control, respectively^38^.

### Characteristics of EPS-1

The phenol-sulfuric acid method was employed to measure the total polysaccharide content of the purified EPS-1^39^,^40^,^41^. HPLC was used to conduct qualitative analysis of the monosaccharide composition of EPS-1. D-fructose, D-glucose, D-xylose, L-rhamnose, lactose, galactose, mannose, and maltose were used as the standards (these standards were all analytical reagents and purchased from Sinopharm Chemical Reagent Co., Ltd., China). A HPLC Carbohydrate Analysis Column (Aminex^®^ HPX-87P) and a refractive index detector were used for detection, with 10 μL of EPS-1 solution (5 mg/mL) loaded and the following detection conditions applied: distilled water with a flow rate of 0.6 mL/min was used as the mobile phase, and the column temperature was set as 80 °C. The protein concentration of the purified EPS was determined using a Bradford assay with bovine serum albumin as a standard (Bio-Rad)^42^,^43^. Microspectrophotometry (Bei Jing Kai Ao K5600, China) was employed to measure the nucleic acid content of the EPS-1^27^. A FTIR spectrometer (Thermo Nicolet AVtAR 370, USA) was used to examine the functional groups of EPS-1^44^. An XPS (Thermo Scientific ESCALAB 250Xi, USA) was used to explore the elemental composition and relative contents of EPS-1. The XPS measurement was conducted with an ESCALAB MK II electron spectrometer using a monochromated Al KR X-ray source at a base pressure below 5 × 10^−8^ Torr. Scanning was performed over a wide binding energy range (0–1100 eV)^22^. The zeta potentials of the EPS-1 solution (0.05 g/L, pH 2.08), blank kaolin clay solution (10 g/L, pH 2.08), blank *M. aeruginosa* culture (1 × 10^7^ cells/mL, pH 4.0), kaolin flocculating system solution, and *M. aeruginosa* flocculating system solution were analyzed using a Zeta Potential Analyzer (Zetasizer Nano-zs 90, Malvern, Co., UK)^45^.

### Sodium Dodecyl Sulfate-Poly Acrylamide Gel Electrophoresis (SDS-PAGE)

The protein composition in EPS-1 and molecular weights of proteins were analyzed using a vertical plate type electrophoresis tank (VE180, Tanon, Shanghai, China)^46^. The 5% stacking gel consisted of 2.03 mL ddH_2_O, 0.84 mL 4 × upper buffer, 0.5 mL30% acrylamide, 2.5 μL N,N,N′,N′-tetra ethylene dimethylene diamine (TEMED), and 25 μL 10% APS. The resolving gel consisted of 2.5 mL ddH_2_O, 1.8 mL 4 × lower buffer, 2.9 mL 30% acrylamide, 5 μL TEMED, and 50 μL 10% APS. The running buffer consisted of 0.25 M Tris (pH 8.3), 0.19 M glycine, and 0.4% SDS. A total of 40 μL of EPS-1 solution (0.05 g/L) was incubated with 10 μL of loading buffer (0.125 M Tris-Buffer (pH 6.8), 4% SDS, 20% glycerol, 10% *β*–mercaptoethanol), boiled (10 min), and then finally loaded (20 μL) in the wells of the gel and subjected to electrophoresis (voltage: 100 V; time: 90 min). The gel was stained in Coomassie Brilliant Blue R-250 solution (0.1% CBB, 50% absolute ethyl alcohol, and 10% glacial acetic acid) by heating until it boils at a time interval of 10 s, and then repeatedly heated 5 times in an oven; it was then de-stained (with 10% absolute ethyl alcohol and 10% glacial acetic acid) overnight in an orbital shaker (ZD-9556, HLD Laboratory Equipment Co., Ltd., Guangzhou, China). The protein bands were then visualized and photographed using a camera (WB100, Samsung, Korea).

### Evaluation of the relative flocculation contribution of proteins in the EPS-1

EPS-1 solutions at a concentration of 0.05 g/L were subjected to two treatment conditions: high temperature treatment at 100 °C for 30 min and exposure to proteinase K (300 μg/mL) at 37 °C for 3 h. The flocculating activities against kaolin suspension (10 g/L, pH = 2.08) of these treated EPS-1 solutions were measured within 10 min using the method described in the ‘*Measurement of flocculating activity’* section. The decrease in the total flocculating activity of EPS-1 after two different treatments was used to evaluate the relative contribution of the protein components in the EPS-1 to its flocculation activity.

### Source of M. aeruginosa

We purchased the *M. aeruginosa* FACHB-905 strain from the Institute of Hydrobiology, Chinese Academy of Sciences, Wuhan, China, and cultured it in Blue-Green 11 medium at 25 ± 1 °C and 3000 Lux, with a light:dark period of 12:12 h, for 7 days^27^.

### Optimization of flocculation parameters of EPS-1 for kaolin suspension and M. aeruginosa removal

The 3-level-3-factor Box-Behnken Design (BBD), a standard RSM, was employed to evaluate the most important operating variables (CaCl_2_ (X_1_), pH (X_2_), and EPS-1 dosage (X_3_)) in the flocculating process; simultaneously, a model was developed based on the equation of the BBD^47^,^48^,^49^,^50^. Based on the results of a preliminary experiment, the ranges of the variables were chosen as follows: for the kaolin suspension, EPS-1 dosage was 0.05–0.2 g/L, CaCl_2_ was 5–15 g/L, and pH was in the range of 2–4; for *M. aeruginosa*, EPS-1 dosage was 0.1–0.4 g/L, CaCl_2_ was 5–15 g/L, and pH was in the range of 4–6 (see detailed information in Table 4). In this study, 15 trials were performed and the independent variables were investigated at three different levels: low level (−1), medium level (0), and high level (+1). These values and data from the experimental design are shown in Table 4. The average flocculating activities obtained in these trials were used as the response variable (Y), and all the experiments were carried out in triplicate.

The response variable (Y) was fitted to a second-order model containing the independent variables of the form of the second-degree polynomial equation stated below:


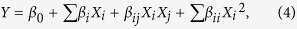


where *Y* is the predicted response, *β*_*0*_ is the intercept, *β*_*i*_ is the linear coefficient, *β*_*ii*_ is the quadratic coefficient, *β*_*ij*_ is the linear-by-linear interaction between the *X*_*i*_and *X*_*j*_ regression coefficients, and *X*_*i*_and *X*_*j*_ are input variables that influence the response variable *Y*.

### Flocculating properties of EPS-1 against kaolin and M. aeruginosa under the optimal parameters

For *M. aeruginosa* removal, EPS-1 solution was first prepared by dissolving 0.24 g of EPS-1 in 1 L distilled water. The flocculating activity of the bioflocculant against *M. aeruginosa* (FA_3_) was then evaluated. In the experiment, the pH of *M. aeruginosa* culture was adjusted to 4.0 and its cell concentration was adjusted to 1 × 10^7^ cells/mL. A mixture containing 30 mL of the aforementioned *M. aeruginosa* culture, 1 mL of EPS-1 solution, and 1 mL of CaCl_2_ solution (5 g/L) was produced in a 100 mL beaker. The reaction vessel was mixed (340 × *g*) for 2 min before the OD at 680 nm of the upper phase was measured with a spectrophotometer (7230G, Shanghai, China) for 30 min. FA_3_ was calculated according to Eq. 3. *M. aeruginosa* culture (30 mL) mixed only with 1 mL CaCl_2_ solution under the conditions described above was used as the control.

For kaolin removal, 0.05 g of EPS-1 was first dissolved in 1 L distilled water. The flocculating activity of the bioflocculant against kaolin (FA_4_) was then evaluated. In the experiment, the pH of kaolin suspension with a concentration of 10 g/L was adjusted to 2.08. A mixture containing 30 mL of the aforementioned kaolin suspension, 1 mL of EPS-1 solution, and 1 mL of CaCl_2_ solution (5.04 g/L) was produced in a 100 mL beaker. The reaction vessel was mixed (340 × *g*) for 2 min before the OD at 550 nm of the upper phase was measured with a spectrophotometer (7230G, Shanghai, China) for 30 min. FA_4_ was calculated according to Eq. 3. Kaolin suspension (30 mL) mixed only with 1 mL CaCl_2_ solution under the conditions described above was used as the control.

### Statistical analyses

The model was statistically evaluated using ANOVA. The results of the analysis included Fisher’s F-test (for overall model significance), the associated probability p (F), the correlation coefficient R, and the determination coefficient R^2^, which indicates the goodness of fit of the regression model.

## Conclusion

A novel bioflocculant, EPS-1, was reported in this study. No less than 18 species of proteins were identified in EPS-1 and the total protein content was 31.70%, with the flocculation contribution of the proteins being estimated at no less than 33.93%. Polysaccharides, which occupied a 57.12% proportion of EPS-1 content, consisted of maltose, D-xylose, mannose, and D-fructose. Carbonyl, amino, and hydroxyl groups were predominant in EPS-1, and three elements, C1s, N1s, and O1s, were mainly present in the bioflocculant. The charge neutralization mechanism was found to play an important role in the kaolin flocculation process. The maximum flocculation efficiencies were 93.34% within 2 min for kaolin suspension and 87.98% within 10 min for *M. aeruginosa*. Therefore, this study demonstrates the potential of EPS-1 as a natural bioflocculant for use in treating wastewater and cyanobacteria-polluted freshwater.

## Additional Information

**How to cite this article**: Sun, P. *et al.* Revealing the characteristics of a novel bioflocculant and its flocculation performance in *Microcystis aeruginosa* removal. *Sci. Rep.* **5**, 17465; doi: 10.1038/srep17465 (2015).

## Supplementary Material

Supplementary Information

## Figures and Tables

**Table 1:** Box-Behnken Design arrangement and responses.

Run	Code levels			FR (%) for kaolin suspension		FR (%) for *Microcystis aeruginosa*	
	X_1_	X_2_	X_3_	Actual value	Predicted value	Actual value	Predicted value
1	−1	0	−1	90.2768	90.7624	78.8569	78.0376
2	1	−1	0	21.1022	20.8779	18.7850	17.9321
3	1	0	−1	93.2260	93.1662	81.6147	82.8807
4	0	−1	−1	94.0286	94.3126	82.3741	81.9609
5	0	−1	1	17.3718	18.0816	12.7898	12.8233
6	1	0	1	15.3866	14.9010	10.9113	11.7306
7	0	0	0	21.9456	22.7276	19.2246	19.6376
8	0	1	−1	93.9877	93.2780	81.0552	81.0217
9	0	0	0	19.1278	22.7276	18.9049	19.6376
10	−1	1	0	23.9973	24.2216	20.1039	20.9567
11	1	1	0	25.8084	26.5780	22.5020	21.2695
12	−1	0	1	21.2536	21.3134	18.9729	17.7069
13	0	1	1	22.0790	21.7949	18.2654	18.6786
14	−1	−1	0	28.0125	27.2429	18.1455	19.3780
15	0	0	0	27.1093	22.7276	20.7834	19.6376

**Table 2:** ANOVA for response surface quadratic model.

Source	Sum of Squares	DF	Mean Square	F Value	Prob > F	CoefficientEstimate
For kaolin suspension						
Model	14820.9	9	1646.77	230.63	0	
Residual	35.7	5	7.14			
Lack of Fit	2.9	3	0.98	0.06	0.976	
Pure Error	32.8	2	16.38			
Cor Total	14856.6	14				
R^2^ = 0.9976	Adj. R^2^ = 0.9933	Pred R^2^ = 0.9919	C.V. = 4.42	Adeq Precision = 26.890		
For *Microcystis aeruginosa*						
Model	11695.7	9	1299.53	569.72	0	
Residual	11.4	5	2.28			
Lack of Fit	9.4	3	3.13	3.10	0.254	
Pure Error	2.0	2	1.01			
Cor Total	11707.1	14				
R^2^ = 0.9990	Adj. R^2^ = 0.9973	Pred R^2^ = 0.9868	C.V. = 4.42	Adeq Precision = 26.890		

**Table 3:** Zeta potentials of these four different systems.

Flocculation system	Zeta potential (mV)
Blank kaolin solution	18.5 ± 0.98
Kaolin flocculating system solution	6.13 ± 0.29
Blank *M. aeruginosa* culture	−22 ± 5.16
*M. aeruginosa* flocculating system solution	−21.6 ± 2.91

**Table 4:** Coding and levels of experiment factors.

Factor	symbol	Code levels					
		For kaolin suspension			For *Microcystis aeruginosa*		
		−1	0	1	−1	0	1
CaCl_2_ (g/L)	X_1_	5	10	15	5	10	15
pH	X_2_	2	3	4	4	5	6
EPS-1 (g/L)	X_3_	0.05	0.1	0.2	0.1	0.2	0.4
