# Supplementary material for: Revealing the characteristics of a novel bioflocculant and its flocculation performance in Microcystis aeruginosa removal
Source: Sci Rep. 2015 Dec 2;5:17465. doi: 10.1038/srep17465 (PMC4667227; doi:10.1038/srep17465)
Supplement: Supplementary Information [file srep17465-s1.pdf]

Revealing the characteristics of a novel bioflocculant and its flocculation performance in *Microcystis aeruginosa* removal

Pengfei Sun[a], Cai Hui[a], Naling Bai[a], Shengmao Yang[b], Li Wan[c], Qichun Zhang[d]*, YuHua Zhao[a]*

[a] *College of Life Sciences, Zhejiang University, 310058 Hangzhou, Zhejiang, PR China*

[b] *Institute of Environment Resource and Soil Fertilizer, Zhejiang Academy of Agriculture Science, 310021 Hangzhou, Zhejiang, China.*

[c] *Department of Chemical and Biomolecular Engineering, Vanderbilt University, 37235-1604, Nashville, Tennessee, USA*

[d] *College of Environmental and Resource Sciences, Zhejiang University, 310058 Hangzhou, Zhejiang, PR China*

* Corresponding authors.
Tel.: +86-571-88982413; E-mail address: qczhang@zju.edu.cn (Q.C. Zhang)
Tel. +86-571-88208557; E-mail address: microbeapply@zju.edu.cn (Y.H. Zhao)

1 AAGTATCCGGCGGTCTTCACGGTGTAGGGGCATCCGTCGTAAACGCCTTGTCGACCACTC

61 TTGACGTTACGGTTCATCGTAAGTATCCGGCGGTCTTCACGGTGTAGGGGCATCCGTCGT

121 AAACGCCTTGTCGACCACTCTTGACGTTACGGTTCATCGTGACGGAAAAATCCATTATCA

181 GGCGTACGAGCGCGGTGTACCTGTGGCCGATCTTGAAGTGATCGGCGAAACTGATAAGAC

241 CGGAACGATTACGCACTTCGTTCCGGACCCGGAAATTTTCAAAGAAACAACTGTATATGA

301 CTATGATCTGCTTTCAAACCGTGTCCGGGAATTGGCCTTCCTGACAAAAGGCGTAAACAT

361 CACGATTGAAGACAAACGTGAAGGACAAGAACGGAAAAACGAGTACCACTACGAAGGCGG

421 AATCAAAAGCTATGTTGAGTACTTAAACCGTTCCAAAGAAGTCGTTCATGAAGAGCCGAT

481 TTATATTGAAGGCGAGAAAGACGGCATAACGGTTGAAGTTGCATTGCAATACAACGACAG

541 CTATACAAGCAATATTTATTCTTTCACAAATAATATCAACACATACGAAGGCGGCACGCA

601 CGAGGCCGGATTTAAAACCGGTCTGACCCGTGTCATAAACGACTATGCAAGAAGAAAAGG

661 GATTTTCAAAGAAAATGATCCGAATTTAAGCGGGGATGATGTGAGAGAAGGGCTGACTGC

721 CATTATTTCAATTAAGCACCCTGATCCGCAATTCGAAGGGCAGACGAAAACCAAGCTCGG

781 CAACTCCGAAGCGAGAACGATCACTGATACGCTGTTTTCTTCTGCGCTGGAAACATTCCT

841 TCTTGAAAATCCGGACTCAGCCCGCAAAATCGTTGAAAAAGGTTTAATGGCCGCAAGAGC

901 GCGGATGGCGGCGAAAAAAGCCCGGGAATTGACCCGGCGCAAAAGTGCGCTTGAGATTTC

961 CAATCTGCCGGGCAAACTGGCGGACTGTTCTTCTAAAGATCCGAGCATTTCCGAGCTGTA

1021 TATCGTAGAGGGTGACTCTGCGGGCGGATCAGCGAAACAGGGACGGGACCGTCATTTCCA

1081 AGCCATTCTGCCGCTGCGCGGTAAGATTCTGAACGTTGAGAAAGCCAGACTTGATAAGAT

1141 TCTCTCAAACAATGAGGTCAGATCAATGATCACGGCCCTCGGAACAGGAATCGGAGAAGA

1201 TTTTAATCTTGAAAACGCGCGTTATCATAAAGTGGTC

Figure S1 Sequence of the *gyrB* gene of *Bacillus amyloliquefaciens* DT.

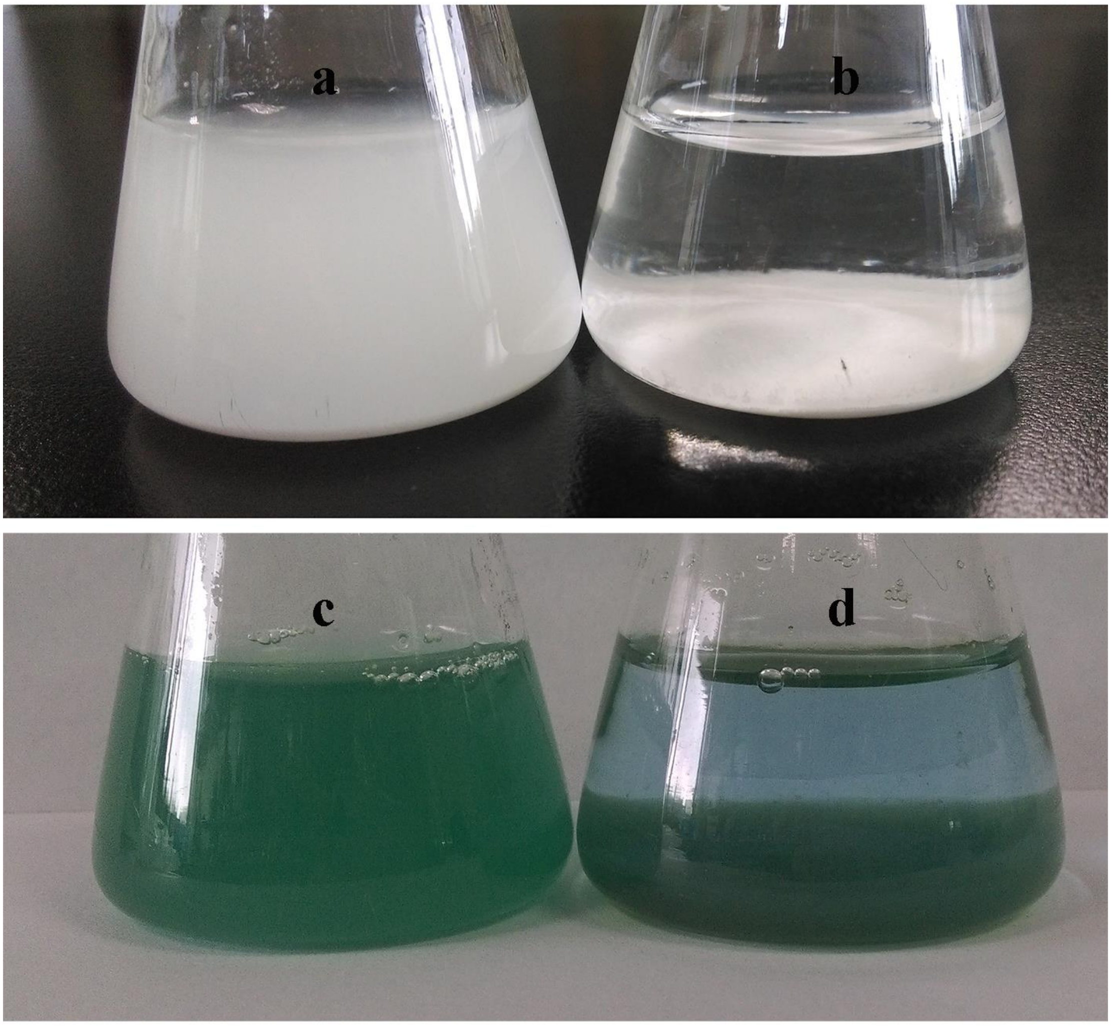

Figure S2 The actual effects of EPS-1 against kaolin suspensions and *Microcystis aeruginosa* cultures. (a) Untreated kaolin suspension; (b) EPS-1 treated kaolin suspension; (c) Untreated *Microcystis aeruginosa* culture; (d) EPS-1 treated *Microcystis aeruginosa* culture.
